# A Comparative Study on the Prediction of Occupational Diseases in China with Hybrid Algorithm Combing Models

**DOI:** 10.1155/2019/8159506

**Published:** 2019-09-29

**Authors:** Yaoqin Lu, Huan Yan, Lijiang Zhang, Jiwen Liu

**Affiliations:** ^1^Department of Occupational and Environmental Health, College of Public Health, Xinjiang Medical University, Wulumuqi, Xinjiang 830011, China; ^2^Xinjiang Engineering Technology Research Center for Green Processing of Nature Product Center, Xinjiang Autonomous Academy of Instrumental Analysis, Urumqi, Xinjiang 830011, China; ^3^Department of Occupational Disease Prevention and Control, Wulumuqi Center for Disease Control and Prevention, Wulumuqi, Xinjiang 830026, China

## Abstract

Occupational disease is a huge problem in China, and many workers are under risk. Accurate forecasting of occupational disease incidence can provide critical information for prevention and control. Therefore, in this study, five hybrid algorithm combing models were assessed on their effectiveness and applicability to predict the incidence of occupational diseases in China. The five hybrid algorithm combing models are the combination of five grey models (EGM, ODGM, EDGM, DGM, and Verhulst) and five state-of-art machine learning models (KNN, SVM, RF, GBM, and ANN). The quality of the models were assessed based on the accuracy of model prediction as well as minimizing mean absolute percentage error (MAPE) and root-mean-squared error (RMSE). Our results showed that the GM-ANN model provided the most precise prediction among all the models with lowest mean absolute percentage error (MAPE) of 3.49% and root-mean-squared error (RMSE) of 1076.60. Therefore, the GM-ANN model can be used for precise prediction of occupational diseases in China, which may provide valuable information for the prevention and control of occupational diseases in the future.

## 1. Introduction

Occupational diseases are any health conditions that are primarily due to exposure to risk factors arising from work-related activities [1]. According to WHO, the occupational population currently accounts for around 50.0% of the global population [2]. ILO reports that 2.34 million deaths were from work-related accidents or diseases worldwide yearly, of which 2.02 million were from work-related diseases. In addition, 160 million people suffer from nonfatal work-related diseases. Occupational diseases have become the leading cause of death among workers [3]. The economic losses caused by work-related diseases and accidents account for 4.0%–6.0% of the gross domestic product of the countries and regions concerned in the world [4]. In 2017, China's total population was 1.39 billion. With the largest labor force in the world, its occupational population was 776 million (55.8%) with 286 million (20.6%) being migrant workers [5]. According to incomplete statistics, about 200 million workers in China are exposed to various occupational hazards. Among them, more than 16 million are workers in toxic and harmful enterprises, involving more than 30 different types of industries [6]. They are exposed to various occupational hazards during the process of occupational activities, which cause occupational health damage and even occupational disease-related death. However, occupational diseases are latent and easily neglected. The number of new occupational diseases was almost tripled from 2003 to 2016, with numbers increasing from 12,511 in 2003 to 31,789 in 2016 in China [7, 8]. It also accounts for an estimated 50,000 to 70,000 deaths and 350,000 new cases of illness each year in the United States [9]. The occupational health problem is worldwide, but relatively more workers are under risk in China due to the relatively larger proportion of the occupational population.

The best way to prevent and control disease is to predict ahead of time. In contrary to the field of medicine where prediction research is well-established [10–13], it is relatively new in the field of occupational health [14–16]. Accurate forecasting of occupational diseases can be achieved by analyzing sufficient historical data. However, data collected by current public health surveillance system do not cover detailed essential information, as it is often difficult to obtain in China and in most of other developing countries. Limited data will affect the establishment of predicting models and result in large prediction bias. Therefore, how to build an accurate predictive model with limited data is very challenging in practice.

A solution on how to use limited data to predict was proposed by Deng in 1982. He established the grey systems theory that shows great capability for studying uncertainty problems with poor information, small sample size, uncertain system, and lack of data. This model focuses on poor information systems with partially unknown information [17]. It has been widely and successfully applied in many fields such as social, scientific, industrial, managerial, agricultural, technological, geological, and medical system [18–36], but it is rarely used in occupational health, especially in the prediction of occupational diseases.

Prediction accuracy comes from appropriate model selection with relative features. At present, most good prediction models were contributed by data mining methods. Data mining is a popular interdisciplinary scientific research field. It mainly includes mathematics, statistics, computer, and other related disciplines, including statistical sampling, estimation, hypothesis testing, artificial intelligence, machine learning, pattern recognition, modeling technology, model optimization, and visualization technology. It involves statistical methods such as classification, estimation, prediction, association, and clustering. It also requires enough features to build models. Therefore, how to model and forecast with limited data is a challenging task, as in the case of occupational diseases.

In this study, we combined the grey systems theory and machine learning methods to solve this issue. The GM models contain five models: even grey model (EGM), original difference grey model (ODGM), even difference grey model (EDGM), discrete grey model (DGM), and Verhulst. The fitted values from the GM models using occupational diseases data were used as training data to train the machine learning models. Five state-of-art machine learning models were used in this study including K-Nearest Neighbor (KNN), Support Vector Machine (SVM), Random Forest (RF), Gradient Boosting Machine (GBM), and Artificial Neural Network (ANN). To the best of our knowledge, this is the first time that those five hybrid algorithm combing models were used to predict occupational diseases. The effectiveness and applicability of the models were assessed based on its ability to predict the incidence of occupational diseases in China.

## 2. Methodology

### 2.1. Data

Cases of occupational diseases from 2005 to 2017 were obtained from national health commission of the people's republic of China.

### 2.2. Data Normalization

The incidence of occupational disease for year 2006 was the statistical summary of 29 provinces nationwide; however, the cases of occupational diseases from year 2015 to 2017 were the summary of 31 provinces nationwide. The other years were the statistics of 30 provinces across the country. In order to improve the prediction accuracy, we standardize the data by dividing the incidence with the number of provinces for that year, so that the number of occupational diseases in different years during 2005–2017 was comparable.

**Figure 1:**
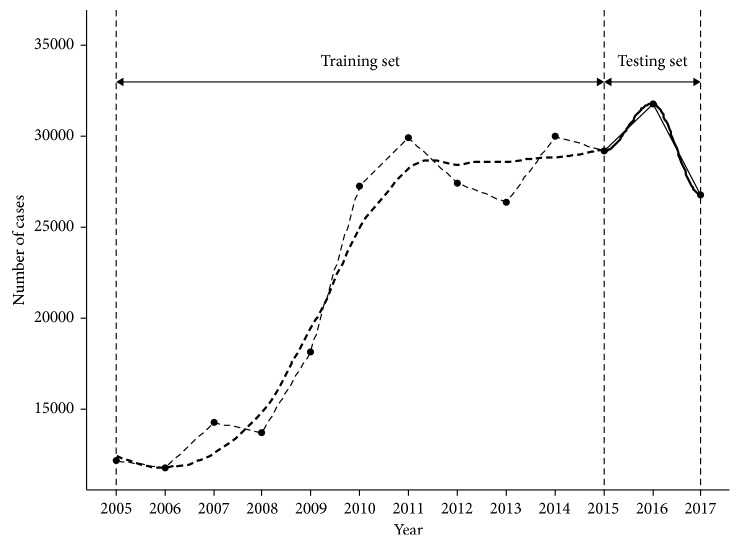
The incidence of occupational diseases in China from 2005 to 2017. The dashed line indicates the first 2/3 of the data used as the training set, and the solid line indicates the last 1/3 of the data used as the testing set. The *Y*-axis represents the number of occupational diseases, and the *X*-axis represents the time series.

Figure 1 illustrates the raw data. The *X*-axis represents the year, and the *Y*-axis represents the number of occupational diseases. We split the data into two parts: the first 2/3 of the data (from 2005 to 2014) were used as the training set and the rest 1/3 were used as the testing set.

### 2.3. Methods

The proposed method was established based on the grey systems theory and the five state-of-art machine learning models, i.e., K-Nearest Neighbor (KNN), Support Vector Machine (SVM), Random Forest (RF), Gradient Boosting Machine (GBM), and Artificial Neural Network (ANN) theory. All the models were run under the R programming language (version 3.6.1).  Table 1 illustrates the models, programming languages, libraries, and parameter adjustment used in this study.

The steps of the hybrid algorithm combing models can be described as follows.

**Step 1 .**

Training the GM models: in order to obtain the training set for the KNN, SVM, RF, GBM, and ANN models, the five GM models, i.e., even grey model (EGM), original difference grey model (ODGM), even difference grey model (EDGM), discrete grey model (DGM), and Verhulst were used to fit the input of the five hybrid algorithm combing models with the training set of China occupational diseases data from 2005 to 2014.

**Step 2 2.**

Training the five hybrid algorithm models: training the KNN, SVM, RF, GBM, and ANN models with different parameters of the training set obtained from step 1 fitting values. Validating the five models with the testing set of the China occupational diseases data from 2015 to 2017.

**Step 3 .**

**Figure 2:**
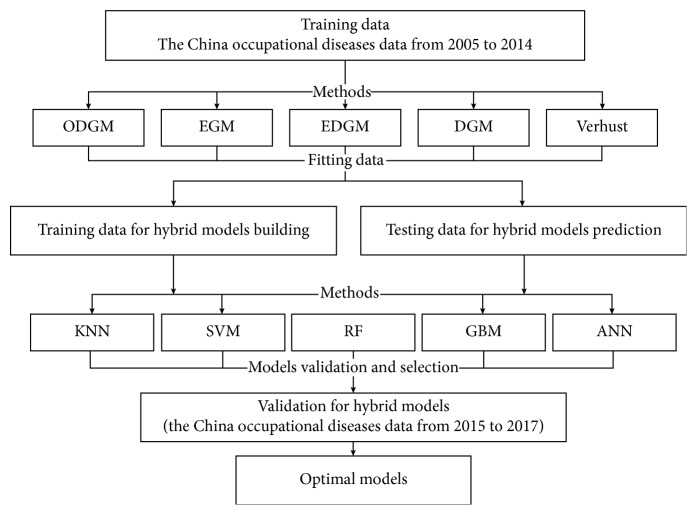
Flowchart of the hybrid method.

Model validation and selection: we compared different models using the mean absolute percentage error (MAPE) and root-mean-squared error (RMSE) as key performance indicators (KPIs). The flowchart of the method is shown in Figure 2.

### 2.4. Metrics

We compared different models using the mean absolute percentage error (MAPE) and root-mean-squared error (RMSE) as key performance indicators (KPIs):

(1) $$\begin{array}{c} \text{MAPE}=\frac{1}{n}\overset{n}{\underset{t=1}{\sum }}\left|\frac{F_{t}-A_{t}}{A_{t}}\right|\times 100\%,\\ \text{RMSE}=\sqrt{\frac{1}{n}\overset{n}{\underset{i=1}{\sum }}{\left(F_{t}-A_{t}\right)}^{2},} \end{array}$$

where *A*_*t*_ is the actual value and *F*_*t*_ is the forecasted value.

## 3. Results

### 3.1. GM Models

Table 2 presents the number of occupational diseases form 2005 to 2017 and the fitted values from the five GM models, respectively.

**Figure 3:**
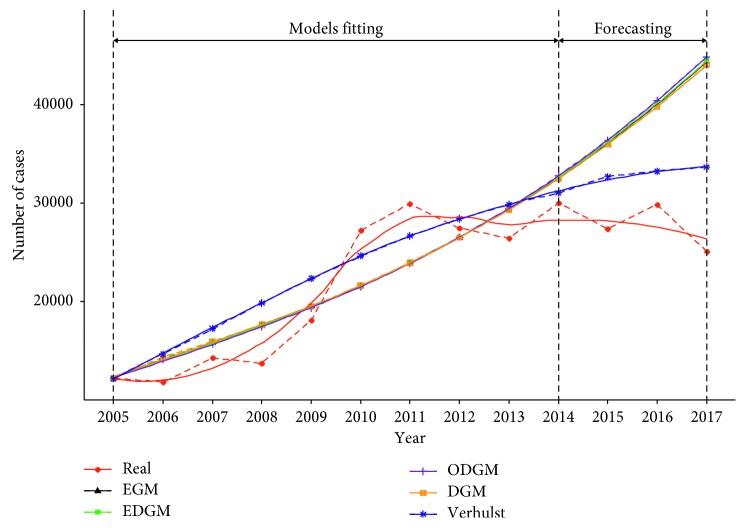
Comparison among real and fitted curves of different grey models for occupational diseases in China.

Figure 3 presents the real values and the fitted values of GM models. Compared to the real values, the fitted values of GM models are not accurate enough although they can predict the general trend.

Table 3 shows the prediction accuracy of GM models. We can see that the MAPE from all GM models are very similar; therefore, in order to keep all information from the original dataset, we adopted all the fitted values as the training set to train the KNN, SVM, RF, GBM, and ANN models.

In order to verify the performance of model selection based on the MAPE and RMSE of the GM models, we selected the training data from the GM models which provides the least MAPE and RMSE values. However, after verification by permutations and combinations, we found that the best model was the one using all the fitted values from the GM models regardless of their MAPE and RMSE values.

This process can be tested with the Occupational Diseases Prediction Online Analysis Platform (http://predict.xjyg.net:666).

### 3.2. GM-KNN Models

**Figure 4:**
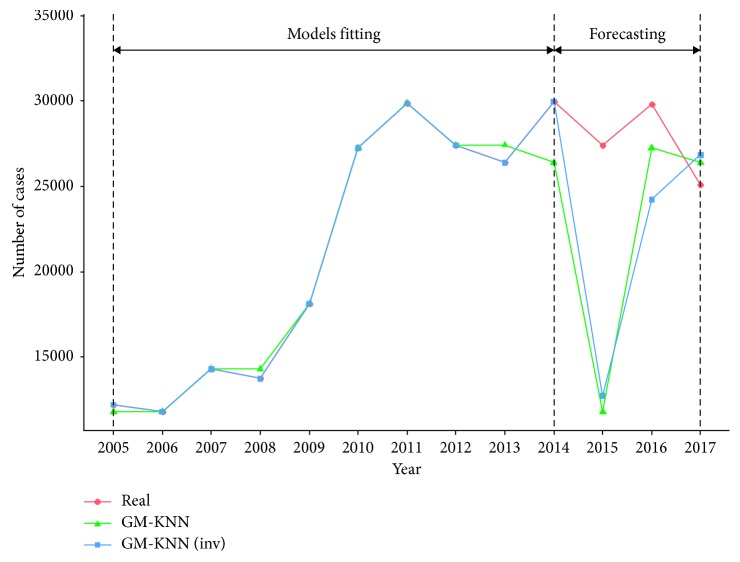
Comparison among real and fitted curves of GM-KNN models.

We used both KNN conventional method and weighted method to build the model, respectively. In the conventional KNN method, we chose the most suitable parameter *k* = 2 for cross-validation. In KNN weighted method, we chose inversion weighting and *k* = 2 for cross-validation and grid scan.  Figure 4 presents the real values and the fitted values of the two GM-KNN models. Compared to the real values, the fitted values of GM-KNN models can predict the general trend well for the training set, but not accurate enough for testing set, so GM-KNN models were not further considered.

### 3.3. GM-SVM Models

**Figure 5:**
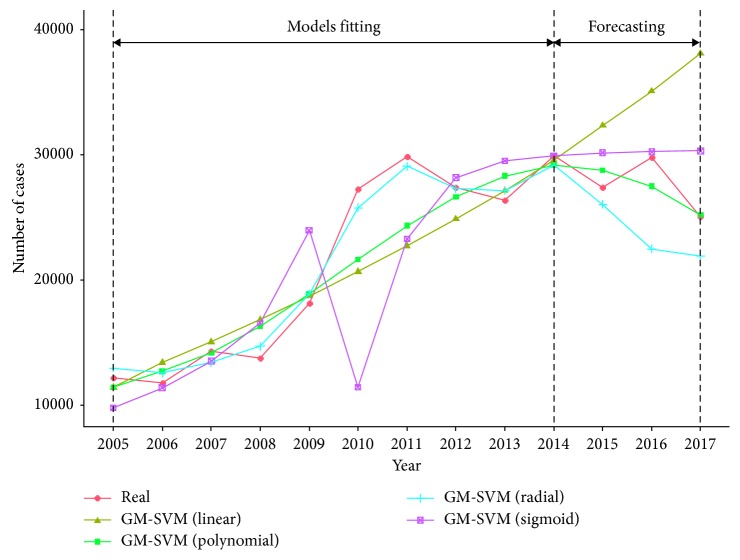
Comparison among real and fitted curves of GM-SVM models.

We built four SVM models with linear, polynomial, radial, and sigmoid kernels, respectively, and the cross-validation method was also applied.  Figure 5 presents the real values and the fitted values of the four GM-SVM models. As shown in Figure 5, the fitted values of the GM-SVM (radial) model showed better fit for the training set, but it predicated much lower values than the real values for the testing set. Among all the models, the predicted values of GM-SVM (polynomial) model were the closest to the real values, but they were still far away from accuracy. Therefore, the GM-SVM models were not considered as good models for prediction.

### 3.4. GM-RF, GM-GBM, and GM-ANN Models

We built the GM-RF model with the optimum parameters of mtry = 1 and ntree = 30 after selecting from 500 trees, the GM-GBM model with *α* = 0.1 and *γ* = 0.5 by the resampling method, and the GM-ANN model with error accuracy of 1 × 10^−8^, 10,000 learning times, and 5 neurons.

**Figure 6:**
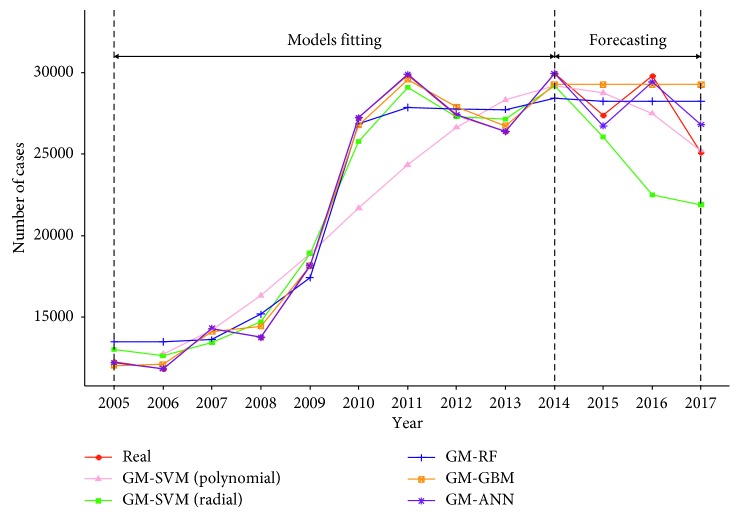
Comparison among real and fitted curves of hybrid models.

Figure 6 presents the fitted values of GM-RF, GM-GBM, and GM-ANN models. Comparing to GM-RF and GM-GBM, the GM-ANN model has the best fit with the fitted and forecasting values being closest to the real values. In addition, GM-ANN model achieved the lowest mean absolute percentage error (MAPE) (3.49%) and the lowest RMSE (1076.60) among all the models (Table 4).

## 4. Discussion

GM models contain five models, and they are even grey model (EGM), original difference grey model (ODGM), even difference grey model (EDGM), discrete grey model (DGM), and Verhulst model. ODGM, EDGM, and DGM can accurately simulate the homogeneous exponential sequence. EGM can handle nonexponential growth and oscillation sequences. ODGM, EDGM, and DGM are good at dealing with nonexponential growth and oscillation sequences near homogeneous exponential series [37]. The main purpose of the Velhulst model is to limit the whole development for a real system, and it is effective in describing some increasing processes, such as an S-curve with a saturation region [38]. In order to verify the performance of model selection based on the MAPE and RMSE GM models, we selected the training data from GM models with the least values of MAPE and RMSE. However, after verification with permutations and combinations, we obtained the best model by using all the GM fitted values regardless of their MAPE and RMSE values. In addition, we observed that the MAPE of GM models are very similar; in order to keep all information of original dataset, we adopted all fitted values as the training set to train the KNN, SVM, RF, GBM, and ANN models.

The results show that GM-KNN models and GM-SVM models are accurate in predicting training set but inaccurate in predicting the testing set. Both Figure 3 and Table 3 show that the MAPE and RMSE of the GM models for the testing data were smaller than those of training data; however, the prediction of the GM-KNN and GM-SVM models was not accurate in predicting the testing data. This phenomenon needs to be further studied.

Although the GM-RF and GM-GBM models achieved lower MAPE (6.99%, 8.45%) and RMSE (2090.13, 2661.27) and their forecasting values were following the general trend and the closest to the real values, the fitted values of these two models were not accurate enough when compared to the real values. GBM is a machine learning technique widely used for regression and classification problems. It produces a prediction model in the form of an ensemble of weak prediction models, typically decision trees. Similar to other boosting methods, it builds the model in a stagewise fashion and generalizes them by allowing optimization of an arbitrary differentiable loss function. Both GBM and RF models demonstrate good performances in big data mining, but they need enough training data to train the model to achieve good predictions. In our study, we only used 10 years of data as the training set; therefore, the model may have been under fitting, which may be the main reason for the inaccurate prediction of the testing set using these two models.

ANN is one of the main tools used in machine learning. It is composed of input and output layers, as well as a hidden layer consisting of units that transform the input into information that the output layer can use. Similar to the synapses in a biological brain, ANN is based on a collection of connected units or nodes called artificial neurons that can transmit signal from one artificial neuron to another. Although ANN are excellent tools for finding patterns that are far too complex, the main issue is that the neural networks are “black boxes”, in which the user feeds in data and receives answers without understanding or access to the exact decision making process. This problem is still the orientation that scientists are exploring at present.

Compared to infectious diseases, occupational diseases have different pathogenesis, relatively few cases, and no obvious seasonal and periodic time series attributes. During the process of disease monitoring, data of occupational diseases generally do not cover the detailed essential information except the collection of the number of cases. It is difficult to build predictive models such as time series model and machine learning models with the limited information. Therefore, Grey model is the best choice for prediction with poor information, small sample size, uncertain system, and lack of data as in the case of occupational diseases. However, in this study, the Grey model did not show significant predictive power being largely deviated from the actual incidence although it could simulate the general trend of incidences Therefore, it can be concluded that single Grey model cannot predict occupational diseases accurately. In order to make up for this shortcoming, we used the simulation results of the grey models as the training data for the five state-of-art machine learning models (KNN, SVM, RF, GBM, and ANN). By comparing to the actual situation, we found that hybrid algorithm combing models performed much better than the single Grey model, where the GM-ANN model had the best performance and achieved the lowest mean absolute percentage error (MAPE) of 3.49% and root-mean-squared error (RMSE) of 1076.60.

In the field of occupational disease, there is no effective predictive method at present. The establishment of hybrid algorithm combing models provides an efficient way for appropriate occupational disease prediction. Most importantly, it provides scientific basis for the prevention and control of occupational diseases and theoretical basis for administrative decision making. It is a scientific method that can be adopted and applied in practical work in the future. It also provides research ideas for other related disciplines.

## 5. Conclusions

In this study, five hybrid algorithm combing models were applied to predict occupational diseases in China. The effectiveness and applicability of the models were assessed based on its ability to predict the incidence trend of occupational diseases in China. To the best of our knowledge, this is the first time that those five hybrid algorithm combing models were used to predict occupational diseases. Through model validation and selection, we found that the GM-ANN model had the best performance and achieved the lowest mean absolute percentage error (MAPE) of 3.49% and root-mean-squared error (RMSE) of 1076.60. Therefore, the precise prediction of the occupational diseases with the GM-ANN model may provide valuable information for prevention and control of the occupational diseases in China. However, further studies and validations with more data are needed in order to put this model prediction method for occupational diseases into practical use.

## Figures and Tables

**Table 1:** The models, programming languages, libraries, and parameter adjustments used in this study.

Models	Programming languages	Libraries	Parameters
GM	R (version 3.6.1)	Self-compiled function	EGM
			ODGM
			EDGM
			DGM
			Verhulst

KNN	R (version 3.6.1)	kknn (version 1.3.1)	*k* = 2
		caret (version 6.0–81)	train.kknn()
			kernel = inv

SVM	R (version 3.6.1)	e1071 (version 1.8–8)	Kernel

RF	R (version 3.6.1)	RandomForest (version 4.6–1.4)	mtry = 1
			ntree

GBM	R (version 3.6.1)	xgboost (version 0.82.1)	nrounds
			colsample_bytree
			min_child_weight
			Eta
			Gamma
			Subsample
			max_depth

ANN	R (version 3.6.1)	nnet (version 7.3–12)	Size
			Decay

**Table 2:** The fitted values of GM models.

Year	Number of occupational diseases	EGM	EDGM	ODGM	DGM	Verhulst
2005	12212	12212	12212	12212	12212	12212
2006	11805	14255	14268	14136	14415	14677
2007	14296	15805	15821	15700	15954	17261
2008	13744	17523	17543	17438	17658	19855
2009	18128	19429	19452	19368	19544	22345
2010	27240	21541	21569	21511	21631	24638
2011	29879	23883	23917	23892	23941	26668
2012	27420	26480	26519	26536	26498	28404
2013	26393	29359	29406	29473	29328	29845
2014	29972	32552	32606	32735	32460	31012
2015	27389	36091	36155	36358	35926	32663
2016	29838	40015	40089	40382	39763	33222
2017	25114	44366	44452	44851	44009	33649

**Table 3:** Accuracy of GM models.

Model	ME	RMSE	MAE	MPE	MAPE
EGM_training	−194.98	3301.76	2721.9	−4.14	13.02
EGM_testing	−12710.28	13539.22	12710.28	−47.51	47.51
EDGM_training	−222.41	3303.21	2729.17	−4.26	13.07
EDGM_testing	−12785.03	13612.4	12785.03	−47.79	47.79
ODGM_training	−191.14	3303.79	2711.09	−3.99	12.85
ODGM_testing	−13083.32	13918.45	13083.32	−48.89	48.89
DGM_training	−255.17	3305.4	2748.96	−4.56	13.32
DGM_testing	−12452.21	13271.52	12452.21	−46.56	46.56
Verhulst_training	−1582.72	3212.63	2745.38	−11.26	15.32
Verhulst_testing	−5730.92	6113.1	5730.92	−21.53	21.53

**Table 4:** Prediction accuracy of hybrid models.

Models	Parameter	Group	ME	RMSE	MAE	MPE	MAPE
GM-KNN	*k* = 2	Training	240.70	1197.26	556.50	0.74	2.32
		Testing	5634.33	9151.44	6487.00	20.17	23.57
	kernel = inv	Training	0.00	0.00	0.00	0.00	0.00
		Testing	6305.41	9155.53	7479.90	22.21	26.89

GM-SVM	kernel = linear	Training	1055.45	3388.72	2422.38	1.71	11.25
		Testing	−7738.91	8587.02	7738.91	−29.16	29.16
	kernel = polynomial	Training	731.33	2742.63	1970.80	1.33	8.94
		Testing	280.26	1573.30	1280.50	0.78	4.45
	kernel = radial	Training	−11.44	863.23	805.92	−1.04	4.43
		Testing	3964.53	4693.51	3964.53	14.10	14.10
	kernel = sigmoid	Training	1333.48	5934.06	3859.30	4.08	17.64
		Testing	−2810.06	3422.28	2810.06	−10.79	10.79

GM-RF	mtry = 1	Training	212.67	1317.38	1174.73	−0.45	6.02
	ntree = 30	Testing	−804.74	2090.13	1862.25	−3.44	6.99

GM-GBM	nrounds = 100	Training	5.27	418.30	365.87	−0.23	1.86
	colsample_bytree = 1						
	min_child_weight = 1						
	eta = 0.1	Testing	−1833.39	2661.27	2205.13	−7.21	8.45
	max_depth = 3						
	Subsample = 0.5						
	Gamma = 0.5						

GM–ANN	Size = 5	Training	−3.29	**16.29**	12.03	−0.01	**0.07**
	decay = 1*e* − 08	Testing	−222.60	**1076.60**	914.97	−1.04	**3.49**

*Note*. ME: mean error; MAE: mean absolute error; MPE: mean percentage error; MAPE: mean absolute percentage error.

## Data Availability

The data used to support the findings of this study are obtained from National Health Commission of the People's Republic of China and are included within the article.

## Supplementary Materials

http://predict.xjyg.net:666/ Supplementary Materials Occupational Diseases Prediction Online Analysis Platform (http://predict.xjyg.net:666/): how to model and forecast with limited data is a challenging task, especially in occupational health fields. In order to control and prevent occupational diseases in practical work effectively and scientifically, we have built Occupational Diseases Prediction Online Analysis Platform. We combined the grey systems theory and machine learning method to solve this issue. The GM models contain five models, and they are even grey model (EGM), original difference grey model (ODGM), even difference grey model (EDGM), discrete grey model (DGM), and Verhulst which were the fitted values from occupational diseases as training data to train the machine learning models. They include five state-of-art models: K-Nearest Neighbor (KNN), Support Vector Regression (SVM), Random Forest (RF), Gradient Boosting Machine (GBM), and Artificial Neural Network (ANN). To the best of our knowledge, this is the first time that those five hybrid algorithm combing models are used to predict occupational diseases. The effectiveness and applicability of the models were assessed based on its ability to predict the incidence of occupational diseases in China.
