# Supplementary material for: A Comparative Study on the Prediction of Occupational Diseases in China with Hybrid Algorithm Combing Models
Source: Comput Math Methods Med. 2019 Sep 29;2019:8159506. doi: 10.1155/2019/8159506 (PMC6791229; doi:10.1155/2019/8159506)
Supplement: http://predict.xjyg.net:666/ Supplementary Materials — Occupational Diseases Prediction Online Analysis Platform (http://predict.xjyg.net:666/): how to model and forecast with limited data is a challenging task, especially in occupational health fields. In order to control and prevent occupational diseases in practical work effectively and scientifically, we have built Occupational Diseases Prediction Online Analysis Platform. We combined the grey systems theory and machine learning method to solve this issue. The GM models contain five models, and they are even grey model (EGM), original difference grey model (ODGM), even difference grey model (EDGM), discrete grey model (DGM), and Verhulst which were the fitted values from occupational diseases as training data to train the machine learning models. They include five state-of-art models: K-Nearest Neighbor (KNN), Support Vector Regression (SVM), Random Forest (RF), Gradient Boosting Machine (GBM), and Artificial Neural Network (ANN). To the best of our knowledge, this is the first time that those five hybrid algorithm combing models are used to predict occupational diseases. The effectiveness and applicability of the models were assessed based on its ability to predict the incidence of occupational diseases in China. [file 8159506.f1.pdf]

Occupational Diseases Prediction Online Analysis Platform

(http://predict.xjyg.net:666/)

1. Introduction

How to model and forecast with limited data is a challenging task, especially in occupational health fields. In this online platform, we combined the grey systems theory and machine learning method to solve this issue. The GM models contain five models and they are even grey model (EGM), original difference grey model (ODGM), even difference grey model (EDGM), discrete grey model (DGM) and Verhulst which was the fitted value from occupational diseases as training data to train the machine learning models. They include five state-of-art models: K-Nearest Neighbor (KNN), Support Vector Regression (SVM), Random Forest (RF), Gradient Boosting Machine (GBM), and Artificial Neural Network (ANN). To the best of our knowledge, this is the first time that those five hybrid algorithm combing models are used to predict occupational diseases. The effectiveness and applicability of the models were assessed based on its ability to predict the incidence of occupational diseases in China.

2. Platform Function

2.1 Data Set

The data set of platform is the cases of occupational diseases from 2005 to 2017 which obtained from national health commission of the people's republic of China.

Data Set module will show the dataset to be used to analysis on the platform. Users can look through the fields of the data set and search the values from data set, that will let users fully understand the structure of data set here (Figure 1).

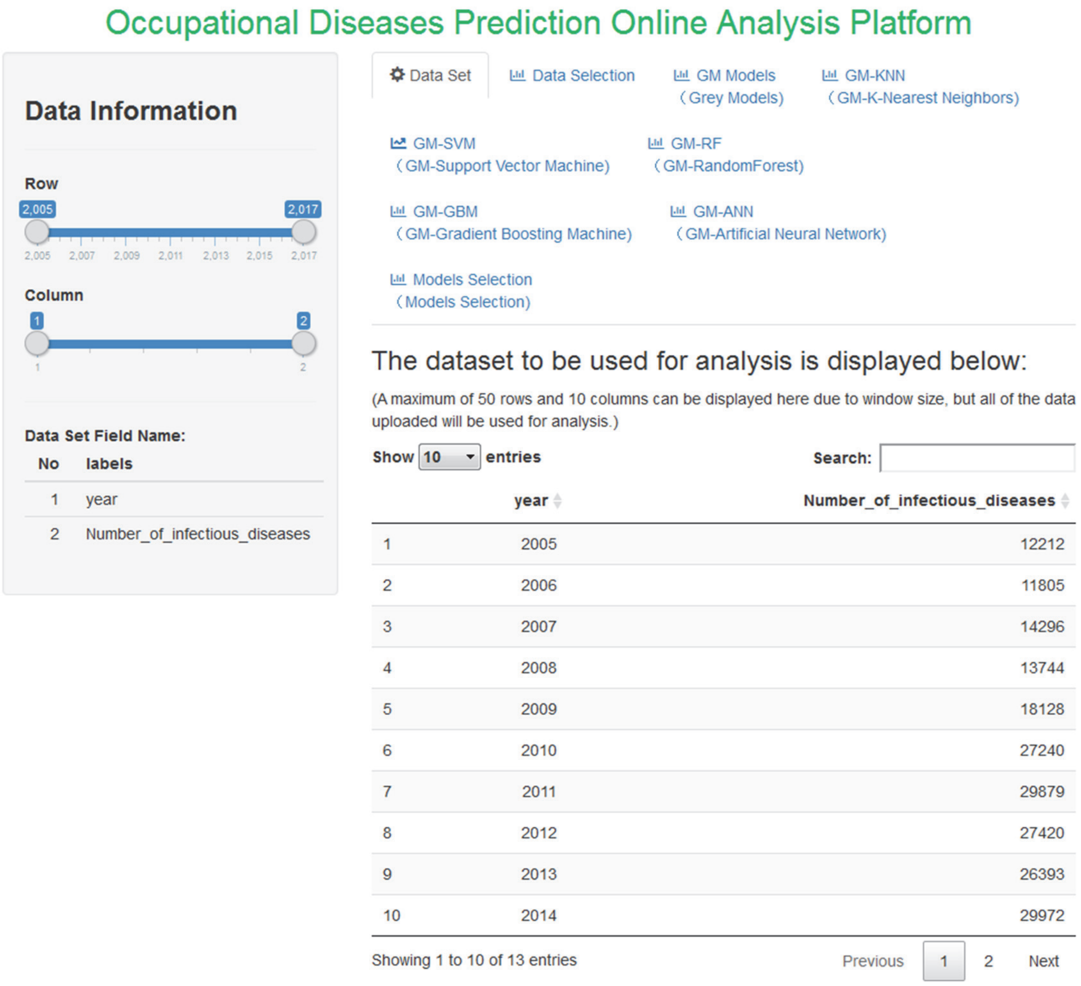

Figure 1. Data set Module

2.2 Data Selection

Data Selection module can select the data range for analysis, in order to give users a good experience, the default training data is from 2005 to 2014 that according to the paper of a comparative study on the prediction of occupational diseases in China with hybrid algorithm combing models. Users can see both plot of the dataset and the specific data set structure (Figure 2).

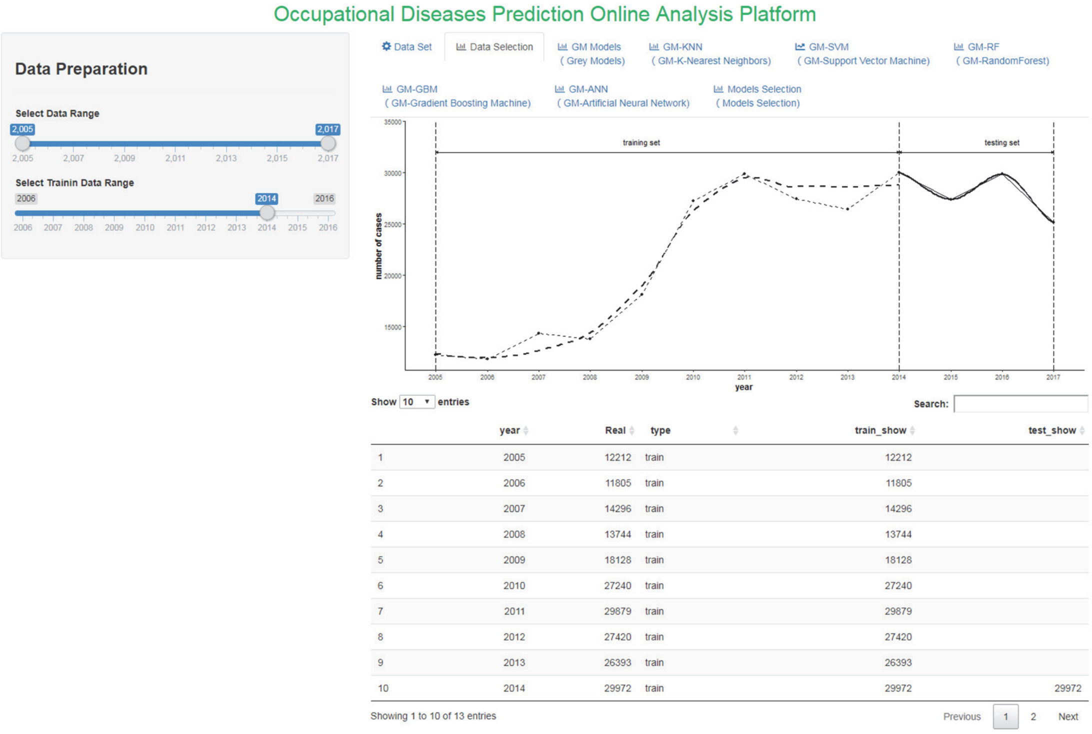

Figure 2. Data Selection Module

2.3 GM Models

In order to obtain the training set for the KNN, SVM, RF, GBM and ANN models, the five GM models, i.e., even grey model (EGM), original difference grey model (ODGM), even difference grey model (EDGM), discrete grey model (DGM) and Verhulst were used to fit the input of the five hybrid algorithm combing models with the training set of China occupational diseases data from 2005 to 2014.

In order to verify the performance of selection by GM model's MAPE and RMSE, we can select the training data by minimum value of GM model's MAPE and RMSE, after verified by permutations and combination, we get the best model was built by all GM fitted values(Figure 3).

Users can see plot of GM models, prediction accuracy and fitted value here. It will update the data automatically according to user's choice.

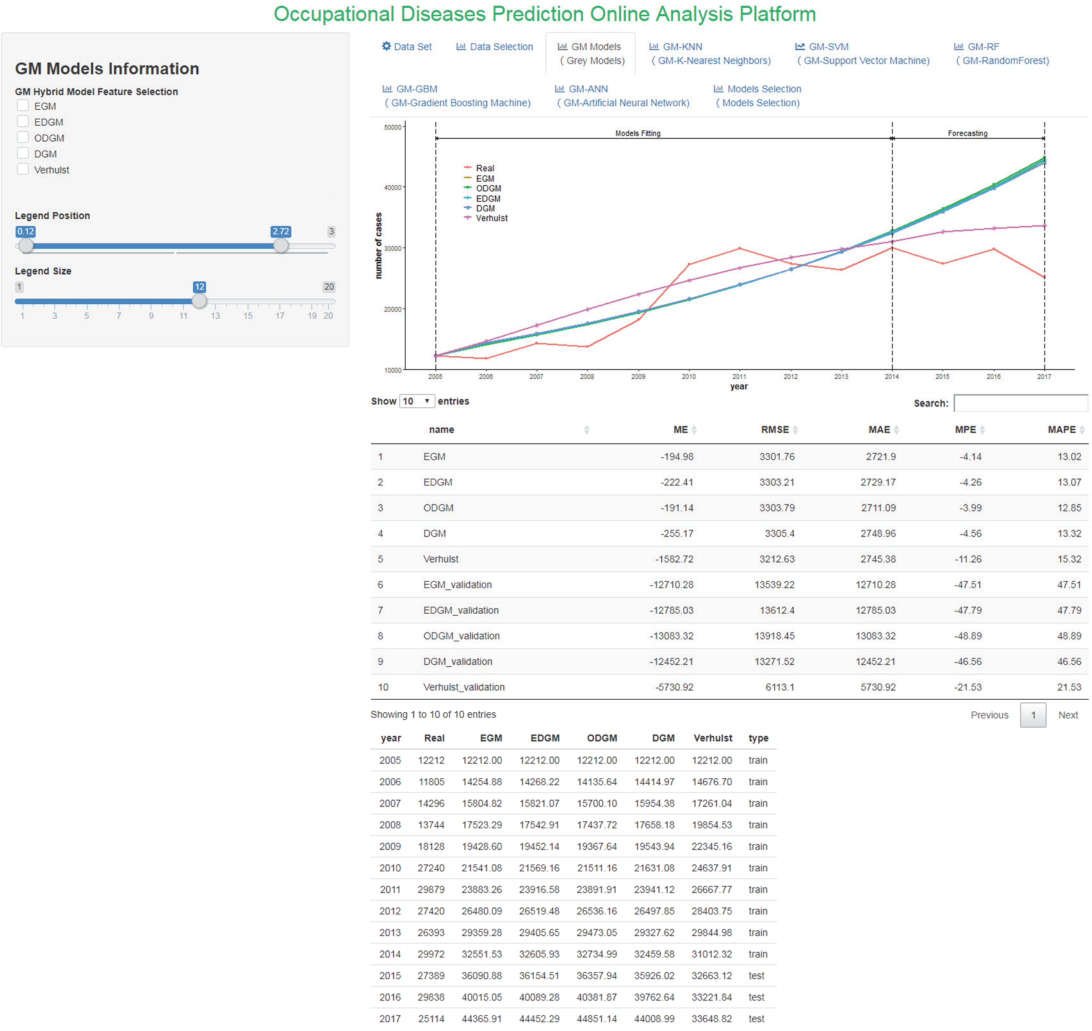

	name	ME	RMSE	MAE	MPE	MAPE
1	EGM	-194.98	3301.76	2721.9	-4.14	13.02
2	EDGM	-222.41	3303.21	2729.17	-4.26	13.07
3	ODGM	-191.14	3303.79	2711.09	-3.99	12.85
4	DGM	-255.17	3305.4	2748.96	-4.56	13.32
5	Verhulst	-1582.72	3212.63	2745.38	-11.26	15.32
6	EGM_validation	-12710.28	13539.22	12710.28	-47.51	47.51
7	EDGM_validation	-12785.03	13612.4	12785.03	-47.79	47.79
8	ODGM_validation	-13083.32	13918.45	13083.32	-48.89	48.89
9	DGM_validation	-12452.21	13271.52	12452.21	-46.56	46.56
10	Verhulst_validation	-5730.92	6113.1	5730.92	-21.53	21.53

year	Real	EGM	EDGM	ODGM	DGM	Verhulst	type
2005	12212	12212.00	12212.00	12212.00	12212.00	12212.00	train
2006	11805	14254.88	14268.22	14135.64	14414.97	14676.70	train
2007	14296	15804.82	15821.07	15700.10	15954.38	17261.04	train
2008	13744	17523.29	17542.91	17437.72	17658.18	19854.53	train
2009	18128	19428.60	19452.14	19367.64	19543.94	22345.16	train
2010	27240	21541.08	21569.16	21511.16	21631.08	24637.91	train
2011	29879	23883.26	23916.58	23891.91	23941.12	26667.77	train
2012	27420	26480.09	26519.48	26536.16	26497.85	28403.75	train
2013	26393	29359.28	29405.65	29473.05	29327.62	29844.98	train
2014	29972	32551.53	32605.93	32734.99	32459.58	31012.32	train
2015	27389	36090.88	36154.51	36357.94	35926.02	32663.12	test
2016	29838	40015.05	40089.28	40381.87	39762.64	33221.84	test
2017	25114	44365.91	44452.29	44851.14	44008.99	33648.82	test

Figure 3. GM Models Module

2.4 GM-KNN (GM-K-Nearest Neighbor)

Platform used both KNN conventional method and weighted method to build the model respectively. In the conventional KNN method, we choose the most suitable parameter k=2 for cross-validation. In KNN weighting method, we choose inversion weighting and k=2 for cross-validation and grid scan.

Users can see plot of GM-KNN models, prediction accuracy and fitted value here. It will update the data automatically according to user's choice (Figure 4).

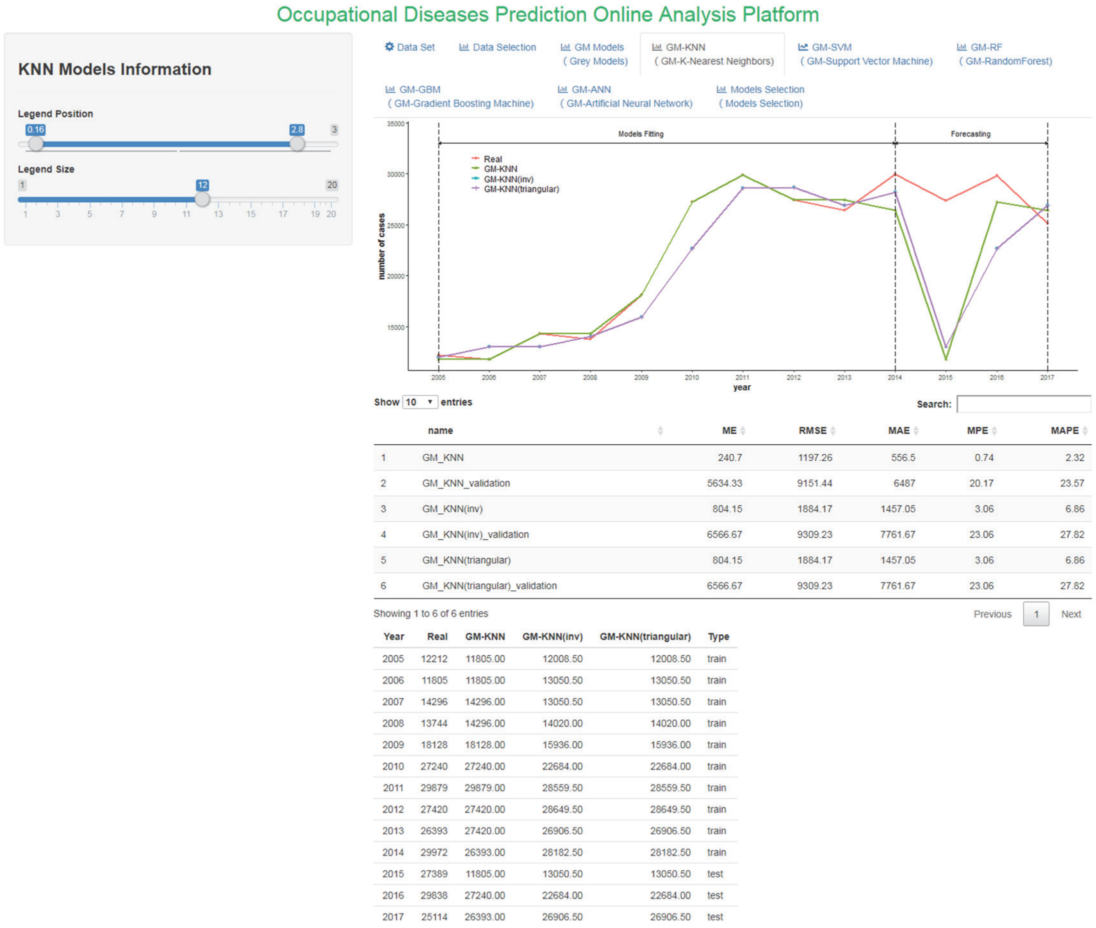

	name	ME	RMSE	MAE	MPE	MAPE
1	GM_KNN	240.7	1197.26	556.5	0.74	2.32
2	GM_KNN_validation	5634.33	9151.44	6487	20.17	23.57
3	GM_KNN(inv)	804.15	1884.17	1457.05	3.06	6.86
4	GM_KNN(inv)_validation	6566.67	9309.23	7761.67	23.06	27.82
5	GM_KNN(triangular)	804.15	1884.17	1457.05	3.06	6.86
6	GM_KNN(triangular)_validation	6566.67	9309.23	7761.67	23.06	27.82

Year	Real	GM-KNN	GM-KNN(inv)	GM-KNN(triangular)	Type
2005	12212	11805.00	12008.50	12008.50	train
2006	11805	11805.00	13050.50	13050.50	train
2007	14296	14296.00	13050.50	13050.50	train
2008	13744	14296.00	14020.00	14020.00	train
2009	18128	18128.00	15936.00	15936.00	train
2010	27240	27240.00	22684.00	22684.00	train
2011	29879	29879.00	28559.50	28559.50	train
2012	27420	27420.00	28649.50	28649.50	train
2013	26393	27420.00	26906.50	26906.50	train
2014	29972	26393.00	28182.50	28182.50	train
2015	27389	11805.00	13050.50	13050.50	test
2016	29838	27240.00	22684.00	22684.00	test
2017	25114	26393.00	26906.50	26906.50	test

Figure 4. GM-KNN Module

2.5 GM-SVM (Support Vector Regression)

Platform built four SVM models with linear, polynomial, radial and sigmoid kernel respectively and with the cross-validation method.

Users can see plot of GM- SVM models, prediction accuracy and fitted value here. It will update the data automatically according to user's choice (Figure 5).

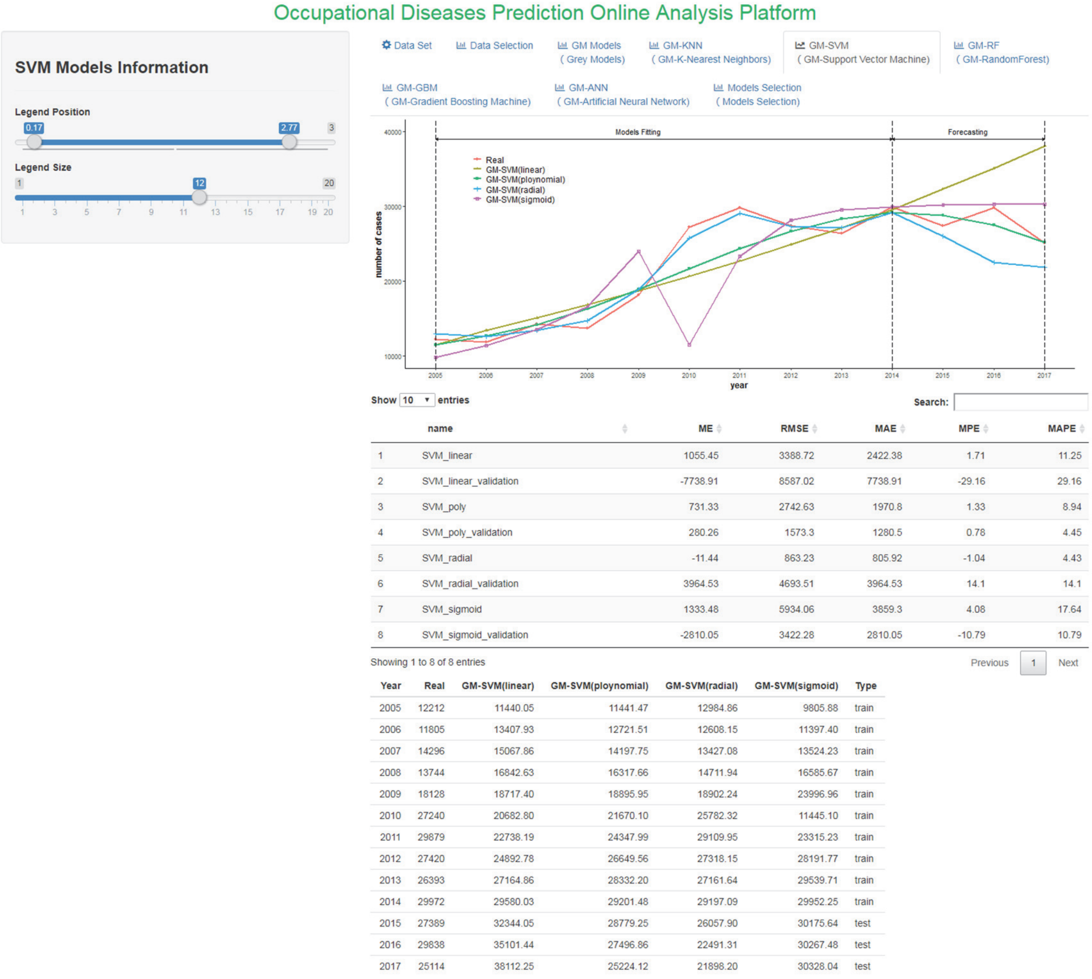

	name	ME	RMSE	MAE	MPE	MAPE
1	SVM_linear	1055.45	3388.72	2422.38	1.71	11.25
2	SVM_linear_validation	-7738.91	8587.02	7738.91	-29.16	29.16
3	SVM_poly	731.33	2742.63	1970.8	1.33	8.94
4	SVM_poly_validation	280.26	1573.3	1280.5	0.78	4.45
5	SVM_radial	-11.44	863.23	805.92	-1.04	4.43
6	SVM_radial_validation	3964.53	4693.51	3964.53	14.1	14.1
7	SVM_sigmoid	1333.48	5934.06	3859.3	4.08	17.64
8	SVM_sigmoid_validation	-2810.05	3422.28	2810.05	-10.79	10.79

Year	Real	GM-SVM(linear)	GM-SVM(ploynomial)	GM-SVM(radial)	GM-SVM(sigmoid)	Type
2005	12212	11440.05	11441.47	12984.86	9805.88	train
2006	11805	13407.93	12721.51	12608.15	11397.40	train
2007	14296	15067.86	14197.75	13427.08	13524.23	train
2008	13744	16842.63	16317.66	14711.94	16585.67	train
2009	18128	18717.40	18895.95	18902.24	23996.96	train
2010	27240	20682.80	21670.10	25782.32	11445.10	train
2011	29879	22738.19	24347.99	29109.95	23315.23	train
2012	27420	24892.78	26649.56	27318.15	28191.77	train
2013	26393	27164.86	28332.20	27161.64	29539.71	train
2014	29972	29580.03	29201.48	29197.09	29952.25	train
2015	27389	32344.05	28779.25	26057.90	30175.64	test
2016	29838	35101.44	27496.86	22491.31	30267.48	test
2017	25114	38112.25	25224.12	21898.20	30328.04	test

Figure 5. GM-SVM Module

2.6 GM-RF (Random Forest)

GM-RF model is built with the optimum parameters of mtry=1 and ntree=30 after selecting from 500 trees.

Users can see plot of GM- RF models, prediction accuracy and fitted value here. It will update the data automatically according to user's choice (Figure 6).

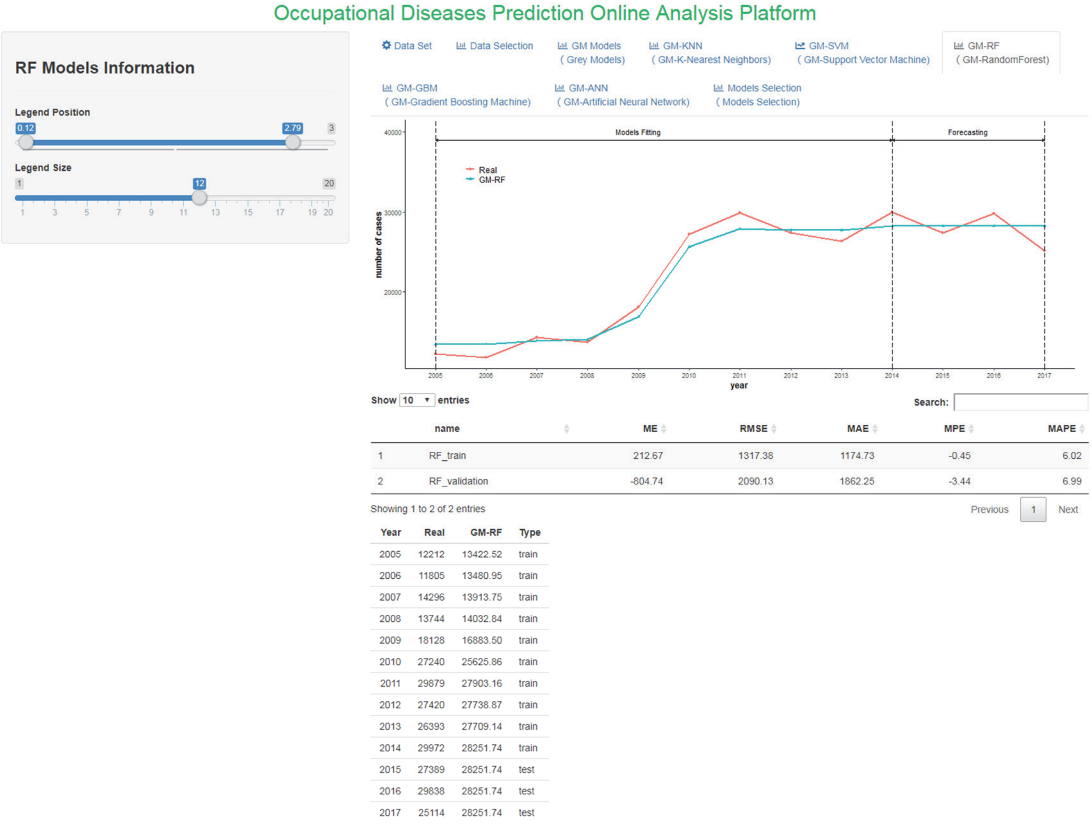

Figure 6. GM-RF Module

2.7 GM-GBM (Gradient Boosting Machine)

GM-GBM model is built with α=0.1and γ=0.5 by resampling method.

Users can see plot of GM- RF models, prediction accuracy and fitted value here. It will update the data automatically according to user's choice (Figure 7).

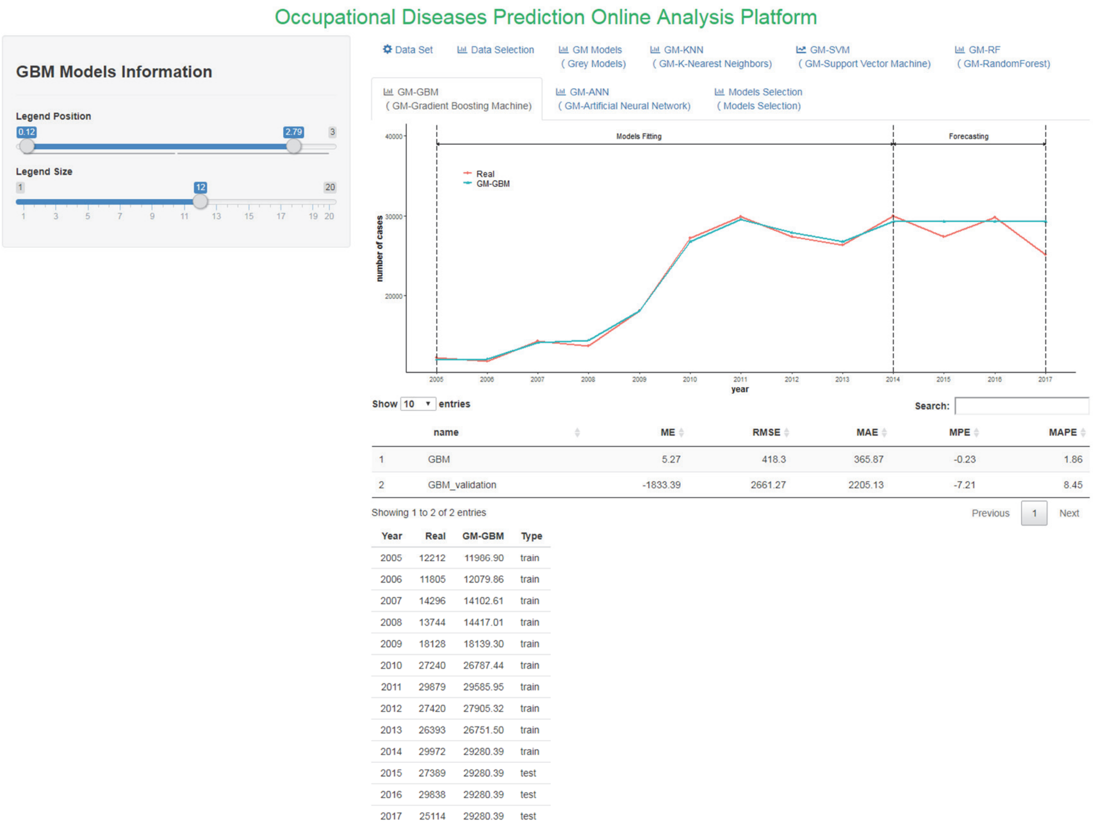

	name	ME	RMSE	MAE	MPE	MAPE
1	GBM	5.27	418.3	365.87	-0.23	1.86
2	GBM_validation	-1833.39	2661.27	2205.13	-7.21	8.45

Year	Real	GM-GBM	Type
2005	12212	11986.90	train
2006	11805	12079.86	train
2007	14296	14102.61	train
2008	13744	14417.01	train
2009	18128	18139.30	train
2010	27240	26787.44	train
2011	29879	29585.95	train
2012	27420	27905.32	train
2013	26393	26751.50	train
2014	29972	29280.39	train
2015	27389	29280.39	test
2016	29838	29280.39	test
2017	25114	29280.39	test

Figure 7. GM-GBM Module

2.8 GM-ANN (Artificial Neural Network)

GM-ANN model is built from error accuracy of 0.00000001, learning times of 10,000 and neurons number of 5.

Users can see plot of GM-ANN model, prediction accuracy and fitted value here. It will update the data automatically according to user's choice (Figure 8).

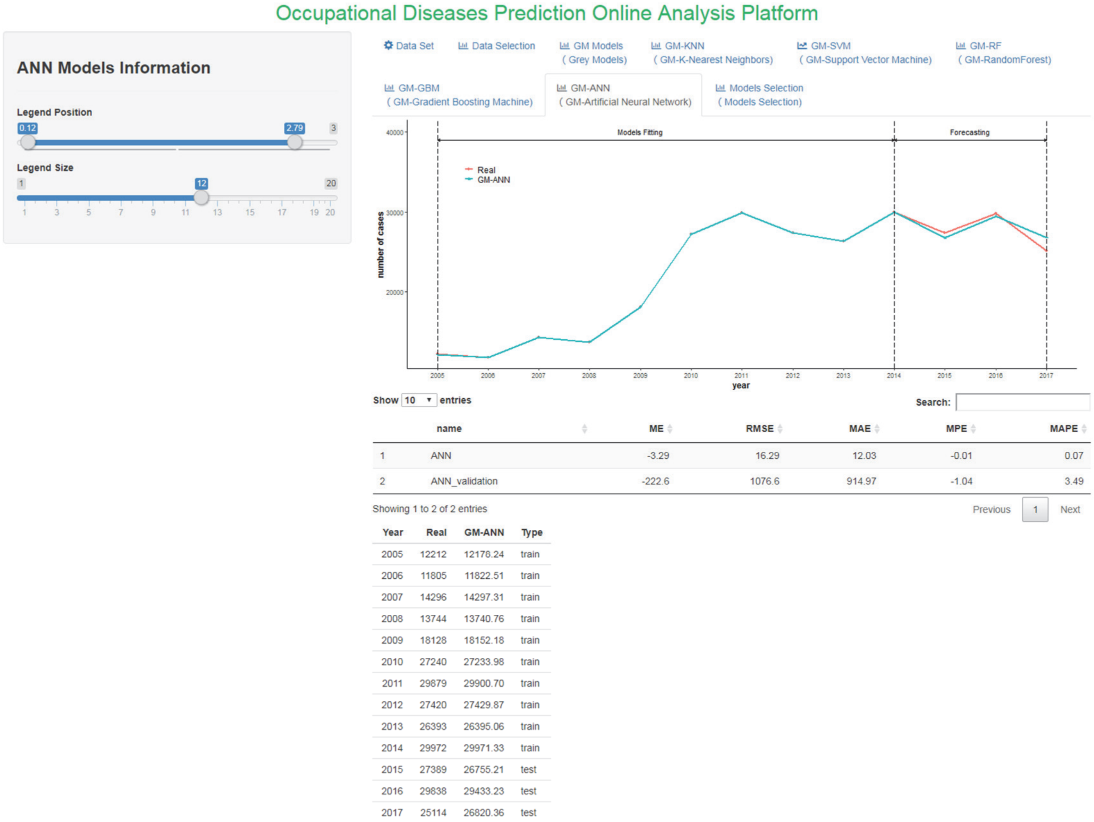

Figure 8. GM-KNN Module

2.9 Models Selection

Users can see plot of GM-KNN, GM-SVM, GM-RF, GM-GBM and GM-ANN models, prediction accuracy and fitted value here. It will update the data automatically according to user's choice (Figure 9).

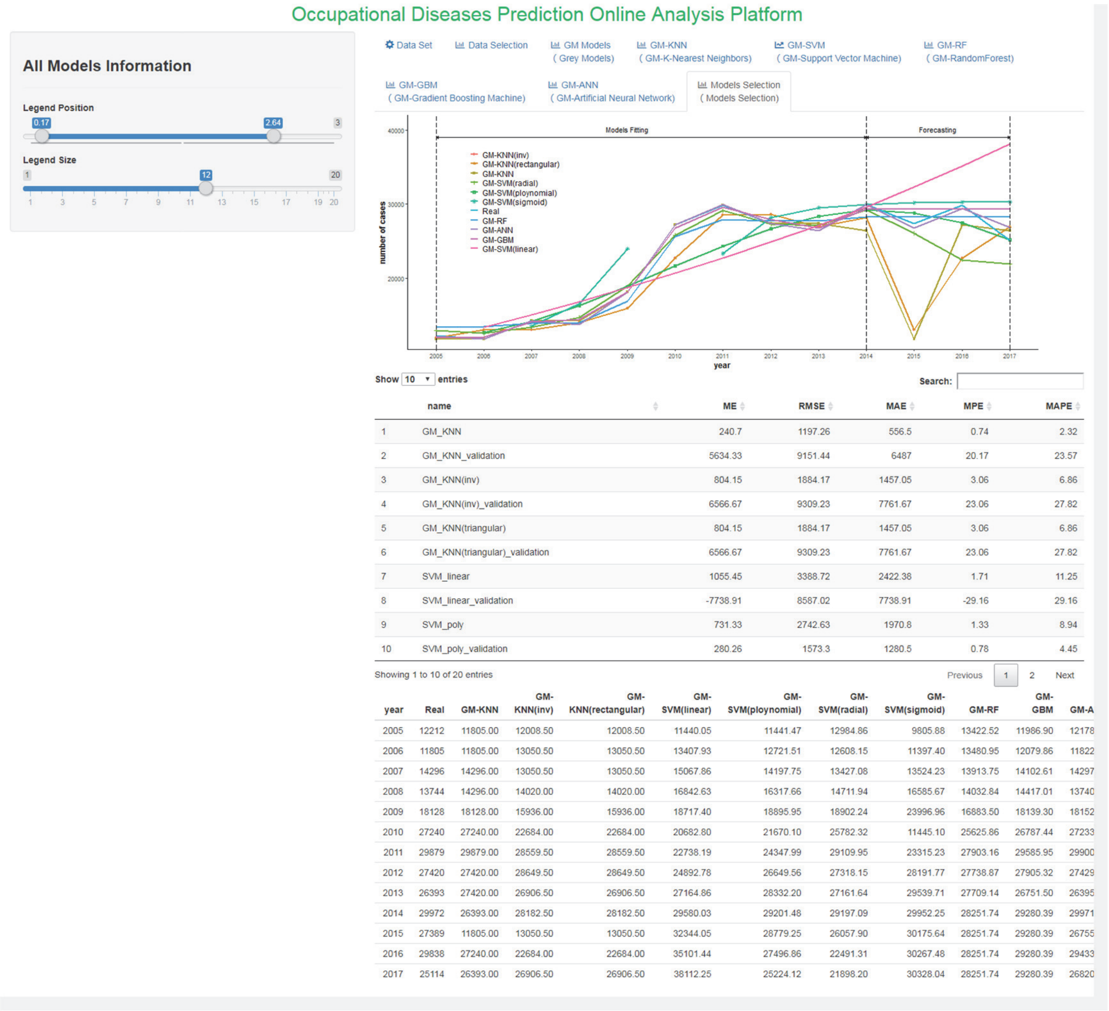

	name	ME	RMSE	MAE	MPE	MAPE
1	GM_KNN	240.7	1197.26	556.5	0.74	2.32
2	GM_KNN_validation	5634.33	9151.44	6487	20.17	23.57
3	GM_KNN(inv)	804.15	1884.17	1457.05	3.06	6.86
4	GM_KNN(inv)_validation	6566.67	9309.23	7761.67	23.06	27.82
5	GM_KNN(triangular)	804.15	1884.17	1457.05	3.06	6.86
6	GM_KNN(triangular)_validation	6566.67	9309.23	7761.67	23.06	27.82
7	SVM_linear	1055.45	3388.72	2422.38	1.71	11.25
8	SVM_linear_validation	-7738.91	8587.02	7738.91	-29.16	29.16
9	SVM_poly	731.33	2742.63	1970.8	1.33	8.94
10	SVM_poly_validation	280.26	1573.3	1280.5	0.78	4.45

year	Real	GM-KNN	GM-KNN(inv)	GM-KNN(rectangular)	GM-SVM(linear)	GM-SVM(ploynomial)	GM-SVM(radial)	GM-SVM(sigmoid)	GM-RF	GM-GBM	GM-A
2005	12212	11805.00	12008.50	12008.50	11440.05	11441.47	12984.86	9805.88	13422.52	11986.90	12178
2006	11805	11805.00	13050.50	13050.50	13407.93	12721.51	12608.15	11397.40	13480.95	12079.86	11822
2007	14296	14296.00	13050.50	13050.50	15067.86	14197.75	13427.08	13524.23	13913.75	14102.61	14297
2008	13744	14296.00	14020.00	14020.00	16842.63	16317.66	14711.94	16585.67	14032.84	14417.01	13740
2009	18128	18128.00	15936.00	15936.00	18717.40	18895.95	18902.24	23996.96	16883.50	18139.30	18152
2010	27240	27240.00	22684.00	22684.00	20682.80	21670.10	25782.32	11445.10	25625.86	26787.44	27233
2011	29879	29879.00	28559.50	28559.50	22738.19	24347.99	29109.95	23315.23	27903.16	29585.95	29900
2012	27420	27420.00	28649.50	28649.50	24892.78	26649.56	27318.15	28191.77	27738.87	27905.32	27429
2013	26393	27420.00	26906.50	26906.50	27164.86	28332.20	27161.64	29539.71	27709.14	26751.50	26395
2014	29972	26393.00	28182.50	28182.50	29580.03	29201.48	29197.09	29952.25	28251.74	29280.39	29971
2015	27389	11805.00	13050.50	13050.50	32344.05	28779.25	26057.90	30175.64	28251.74	29280.39	26755
2016	29838	27240.00	22684.00	22684.00	35101.44	27496.86	22491.31	30267.48	28251.74	29280.39	29433
2017	25114	26393.00	26906.50	26906.50	38112.25	25224.12	21898.20	30328.04	28251.74	29280.39	26820

Figure 9. Models Selection Module
